# Silent Verb Generation during fMRI Reveals Post-Radiotherapy Alterations in Cerebellar-Cerebral Language Regions in Patients Treated for Medulloblastoma

**DOI:** 10.21203/rs.3.rs-9372156/v1

**Published:** 2026-04-14

**Authors:** Josue Luiz Dalboni da Rocha, Ping Zou Stinnett, Stu McAfee, Carolina Torres Rojas, Matthew A. Scoggins, Askery Canabarro, Pankaj Pandey, Thomas Merchant, Giles Robinson, Amar Gajjar, Heather Conklin, Ranganatha Sitaram

**Affiliations:** St. Jude Children Research’s Hospital; St. Jude Children Research’s Hospital; St. Jude Children Research’s Hospital; St. Jude Children Research’s Hospital; St. Jude Children Research’s Hospital; Universidade Federal de Alagoas; St. Jude Children Research’s Hospital; St. Jude Children Research’s Hospital; St. Jude Children Research’s Hospital; St. Jude Children Research’s Hospital; St. Jude Children Research’s Hospital; St. Jude Children Research’s Hospital

**Keywords:** Medulloblastoma, Radiotherapy, fMRI, Cerebellum, Neurocognition

## Abstract

**Background:**

Medulloblastoma is the most common malignant pediatric brain tumor. Although radiotherapy improves survival, it is often associated with neurocognitive impairments affecting language and executive functions in developing brains. We investigated treatment-related changes in task-evoked activation using silent verb generation fMRI.

**Methods:**

Thirty-one children and adolescents with medulloblastoma enrolled on SJMB12 (18 males; mean age 14.1 ± 4.7 years) completed visual and auditory noun-cued covert verb generation during fMRI before radiotherapy and again within 6 weeks after completing radiotherapy. A two-stage, nested feature-selection framework coupled with a linear Support Vector Machine differentiated pre- from post-irradiation scans using leave-one-subject-out cross-validation, benchmarked against a paired pre-to-post label-swap permutation test.

**Results:**

The classifier distinguished pre- from post-irradiation scans with 80.7% leave-one-subject-out accuracy, exceeding the permutation-derived null distribution (one-sided p = 0.0149). Post-treatment task-related BOLD changes showed reduced activity in bilateral cerebellar hemispheres (Crus I/II, lobules VI–VIII), left inferior frontal gyrus, left insula, left putamen, supragenual anterior cingulate, and left middle temporal gyrus. Reduced task-negative responses were observed in a large cluster overlapping right precuneus, right superior parietal, right paracentral, and right postcentral cortices, alongside increased engagement of the left supramarginal gyrus and left inferior parietal lobule.

**Conclusions:**

These findings indicate that radiotherapy is associated with early functional reorganization in cerebellar–cerebral circuits supporting language production and cognitive control, with possible compensatory recruitment of dorsal parietal phonological working memory regions during covert speech. Neuroimaging-based monitoring of these network changes may help guide interventions aimed at minimizing cognitive sequelae in medulloblastoma patients.

## Introduction

Medulloblastoma arises in the posterior fossa and remains the most common malignant pediatric brain tumor, accounting for approximately 20% of all childhood brain malignancies.^1^ Advances in adjuvant therapy, especially risk-adapted radiotherapy have significantly increased survival rates.^2^ However, patients often face a range of life-altering effects, with neurocognitive deficits among the most pronounced and debilitating.^3^ These deficits affect domains such as language, working memory, processing speed, and executive function, ultimately impacting academic achievement and quality of life.^4^ Literature indicates that the developing brain is particularly vulnerable to ionizing radiation, which disrupts proliferative zones and myelination in white matter tracts crucial for language and cognitive functions.^5^

Functional Magnetic Resonance Imaging (fMRI) has emerged as an effective tool for investigating functional alterations in the brain following treatment.^6^ The Blood Oxygenation Level-Dependent (BOLD) contrast provides noninvasive monitoring of changes in local neuronal activity related to specific tasks.^7,8^ Language fMRI paradigms such as silent verb generation and orthographic lexical retrieval typically activate frontal and temporal language regions, as well as the cerebellum.^9^ In particular, the silent verb generation task has demonstrated robust engagement of left frontal and temporal language areas, the supplementary motor area, and, importantly, the cerebellum.^10,11^

The cerebellum is increasingly recognized for its role in higher cognitive functions, including language planning and executive processing.^12,13^ Mounting evidence shows that cerebellar dysfunction can lead to impairment in linguistic, executive, and affective domains.^14,15^ Given these insights, the cerebellum is a pivotal region of interest in understanding the neural underpinnings of post-radiation deficits in medulloblastoma patients.

Despite accumulating research data, our understanding of how radiotherapy specifically modulates language network activation remains incomplete, particularly in younger populations undergoing treatment for medulloblastoma. The current study sought to address this gap by employing fMRI to explore changes in the BOLD signal in a cohort of pediatric medulloblastoma patients assessed before and after radiotherapy. The research had the following objectives:

1. **Characterize** the pattern of task-related brain activation pre- and post-radiotherapy using a silent verb generation paradigm;
2. **Identify** specific regions exhibiting systematic within-subject change in task-evoked BOLD activation after radiotherapy;
3. **Explore** whether machine learning classifiers can effectively differentiate between pre- and post-radiotherapy scans based on activation metrics, thus revealing biomarkers for neurocognitive risk.
4. **Quantify** individual-level expression of the multivariate signature and derive an interpretable percent signal change (PSC) unit network contrast score to summarize treatment-related change.

We hypothesized that radiotherapy would alter task-evoked activation within cerebello-cerebral language and control networks. We further posited that these coordinated changes, captured by multivariate decoding and summarized by clinically interpretable effect metrics, could serve as important early indicators of subsequent neurocognitive deficits.

## Materials and Methods

### Participants

Thirty-one pediatric medulloblastoma patients (18 males; mean age at irradiation: 14.1 ± 4.7 years) were prospectively enrolled on the St. Jude SJMB12 clinical trial (ClinicalTrials.gov identifier: NCT01878617) at a tertiary pediatric oncology center. Participant demographic and clinical characteristics are summarized in Table 1. Inclusion criteria for fMRI were:

1. histologically confirmed medulloblastoma;
2. age greater than or equal to 6 years and less than 22 years at the time of diagnosis;
3. no previous radiotherapy, chemotherapy, or other brain tumor-directed therapy other than corticosteroid therapy and surgery;
4. patients were required to begin treatment as outlined in the protocol within 36 days of definitive surgery (day of surgery is day 0; definitive surgery includes second surgeries to resect residual tumor);
5. Females must not be pregnant or breast-feeding. Female participants > 10 years of age or post-menarche must have a negative serum or urine pregnancy test prior to enrollment;
6. planned standardized post-operative radiotherapy regimen following maximal safe resection;
7. ability to undergo an MRI session without sedation or with minimal sedation; and
8. no contraindications to MRI.

Patients with diagnoses other than medulloblastoma or severe neurologic deficits precluding task participation (e.g., significant aphasia, profound sensorimotor impairment) were excluded. Written informed consent was obtained from legal guardians, and assent was provided by children who met the age and cognitive criteria. This study was approved by the Institutional Review Board (IRB) and conducted in accordance with the Declaration of Helsinki.

### Study Design

All 31 participants underwent fMRI scans pre-radiotherapy (TP1, less than 6 weeks after definitive brain surgery for tumor extraction, but less than 1 week before radiotherapy) and post-radiotherapy (TP2, less than 6 weeks after completing their radiation course, but before initiating chemotherapy). However, only 8 of these 31 participants completed the silent verb generation fMRI protocol at the five protocol-defined visits: TP1 (pre-irradiation baseline), TP2 (post-irradiation and prior to chemotherapy), TP3 (post-chemotherapy; this preceded training for participants enrolled in the cognitive working-memory training, Cogmed trial), TP4 (protocol follow-up visit scheduled after the Cogmed trial period), and TP5 (6-month follow-up after TP4). Cogmed participation was not universal; in the TP1–TP5 subset (n = 8), only 2 participants received the Cogmed intervention (both randomized to training), so TP4 should be interpreted as a protocol timepoint rather than a post-intervention assessment for the full subset. Because the sample size differed markedly from the beginning to the end of the longitudinal data acquisition, the primary analysis focused on the pre- to early post-irradiation contrast (TP1 vs TP2), whereas an exploratory analysis evaluated longitudinal activation score trajectories from TP1 to TP5 in the subset with complete five-visit data. Between TP1 and TP2, patients followed a standard medulloblastoma protocol comprising craniospinal irradiation (15 Gy–39.6 Gy, depending on risk stratification) plus a 51Gy-54Gy boost to the posterior fossa or tumor bed.

### fMRI Task: Silent Verb Generation

Participants completed a silent verb generation task during two separate fMRI runs: one with visually presented nouns and the other with auditory presentation. Each session followed a block design alternating between stimulation periods (for verb generation) and fixation periods (for rest). During stimulation blocks, participants were instructed to silently generate a verb associated with each presented noun (e.g., hearing or seeing “car” and thinking “drive”), participants were instructed to silently think of a verb that could be associated with the noun (e.g., “drive”), without vocalizing their response. Each stimulation block lasted 20 seconds. In the visual modality, nouns were shown on the screen every 3 seconds for 1 second. In the auditory modality, each noun was spoken once during the stimulation period, following the same timing structure. Resting blocks also lasted 20 seconds and displayed a fixation cross continuously.

### Image Acquisition

Images were acquired on a 3T Siemens MRI scanner. High-resolution T1-weighted structural images (voxel size = 0.5×0.5×1 mm^3^) were obtained. Functional data were collected using a gradient-echo echo-planar imaging sequence sensitive to BOLD contrast (TR = 2000 ms, voxel size = 3×3×4mm^3^, matrix size = 64 × 64 × 36). Slices covered the whole brain, including the cerebellum.

### Image Preprocessing

Functional image preprocessing was performed with the SPM software Version 12 (http://www.fil.ion.ucl.ac.uk/spm/, accessed on 27 March 2025).^16^ Steps included:

1. **Realignment**: Motion correction by aligning all functional volumes to a mean reference volume.
2. **Coregistration**: Functional images were co-registered to the respective T1 structural image of each subject.
3. **Normalization**: Spatial normalization to the standard Montreal Neurological Institute (MNI) space.
4. **Segmentation**: Brain tissue and surgical void segmentation to exclude resected tumor areas from spurious signals.
5. **Smoothing**: Gaussian kernel (8 mm FWHM) to enhance signal-to-noise ratio.

### Activation Score Extraction

At the single-subject level, first-level GLMs were constructed to compare task activation during noun presentation blocks (verb generation) versus rest. The “activation score” for each voxel was defined as the t-value derived from the task-versus-rest contrast.

#### Feature Selection Framework.

To isolate the ensemble of voxels that captured treatment-related functional change while rigorously preventing information leakage, we implemented a two-stage, nested feature-selection framework executed independently inside each leave-one-subject-out (LOSO) cross-validation fold. All steps were performed with MATLAB R2024a (MathWorks Inc.).

The first stage removes voxels whose direction of change is inconsistent across patients, thus attenuating noise from heterogeneous surgical cavities and variable hemodynamic baselines. The subsequent statistical pruning attenuates redundancy among highly correlated voxels and focuses the classifier on the smallest, most informative multivariate signature of radiation-induced alteration. Together, the stages realize a balance between neurobiological interpretability, dimensionality reduction, and predictive power, conforming to current best practices for machine-learning analyses of pediatric neuro-oncology imaging data.

### Feature Selection

#### Stage 1 – Consistency-aware search-light masking

1. Sign-consistency filter
2. For every training subject *s* and voxel *v* we computed the signed activation difference
$$\Delta_{s,v}=T_{s,v}^{TP2}-T_{s,v}^{TP1}$$
  where *T* denotes the first-level task-versus-rest *t*-statistic. A voxel was provisionally retained if at least θ × N_train_ subjects shared the same sign of *Δ _s,v_* (positive *or* negative), where θ is the feature threshold. Eight thresholds were evaluated (θ = 0.55,0.60,…,0.90); the threshold that maximized mean classification accuracy across folds was selected a posteriori (see “Classification Analysis”).
3. Search-light construction
4. Each sign-consistent voxel served as the center of a cubic search-light^17^ with a spherical kernel of radius 4 voxels (12 mm in-plane, 16 mm through-plane given the 3 × 3 × 4 mm^3^ functional resolution). Spheres containing fewer than 25% of the theoretical 257 voxels (i.e., < 64 voxels) after the sign filter were discarded to ensure spatial stability.
5. Sphere ranking
6. Within each eligible sphere, we computed a voxel-wise Fisher score^6^ (signal-to-noise ratio) between TP1 and TP2:
$$F_{v}=\frac{\left|\mu_{TP2,v}-\mu_{TP1,v}\right|}{\sigma_{TP2,v}+\sigma_{TP1,v}+\epsilon }$$
  where *μ*_TP*k,v*_ and *σ*_TP*k,v*_ are the mean and standard deviation of the first-level task-versus-rest t-statistic at voxel *v* across training samples in timepoint *k*, and *ϵ* is a small positive constant added as a numerical proxy for an infinitesimal offset to prevent division by zero. The Fisher score was computed only for voxels within the sphere that also satisfied the sign-consistency mask. The sphere score was defined as the mean of *F*_*v*_ across voxels in the sphere. Spheres were ranked by score (descending), and the top 50% were retained.
7. Mask assembly
8. The union of voxels belonging to the retained spheres constituted the feature mask for that fold. This mask typically encompassed < 10% of intracranial voxels, thereby reducing dimensionality by an order of magnitude while preserving spatial contiguity.

All computations in steps 2–4 considered only training data, guaranteeing strict independence from the held-out subject.

#### Stage 2 – Recursive feature elimination with linear Support Vector Machine (SVM)

Within the identified mask described above (see Stage 1), a linear support-vector machine (SVM^18–20^; default C = 1) was trained on the concatenated voxel values from the training data (label 0 = pre-irradiation, 1 = post-irradiation). Feature pruning followed a modified recursive-feature-elimination (RFE) schedule:

1. **Weight ranking**. Absolute SVM weights were used as a proxy for feature importance.
2. **Fractional retention**. Predetermined fractions of the current feature set (100%, 75%, 50%, 25%, 10%, 5%) were evaluated non-sequentially. For each fraction, only the highest-ranked voxels were kept.
3. **Inner validation.** The retained subset was re-evaluated by leave-one-out cross-validation within the training data only. The subset achieving the highest inner-loop accuracy was selected (ties resolved by the smallest feature count).
4. **Final model.** An SVM was retrained on the optimal subset and subsequently applied to the two scans of the held-out subject.

Because both the search-light mask and the RFE subset were derived exclusively from training data in every LOSO iteration, the procedure avoided circularity (“double dipping”) and yielded an unbiased estimate of generalizability.

### Classification Analysis

We used the selected voxels to define regions of interest (ROIs). We implemented a binary (pre-irradiation versus post-irradiation scans) SVM classification (MATLAB’s fitcsvm, linear kernel). We performed leave-one-patient-out cross-validation^21–23^:

1. In each fold, the data from one patient (pre and post scans) formed the test set, and the remaining 30 patients formed the training set.
2. Accuracy, sensitivity, and specificity were computed across folds.

### Cluster-level PSC change (ΔPSC) and statistical testing

For each cluster with at least 100 voxels (*k*) derived from the union of voxels selected across leave-one-subject-out folds at the optimal consistency threshold (θ = 0.70), we computed mean task-evoked BOLD PSC within the cluster mask for each participant at pre- and post-radiotherapy visits. Within-subject change was defined as Δ PSC (k) = *PSC* (*k*)_TP2_ − *PSC* (*k*)_TP1_. For each cluster, we tested whether the median ΔPSC(k) differed from zero using a two-sided Wilcoxon signed-rank test. Wilcoxon effect size was quantified as r = |z|/√N, where z is the normal-approximation statistic from the signed-rank test and N is the number of paired observations. P-values were adjusted for multiple comparisons within clusters using the Benjamini–Hochberg false discovery rate (FDR)^24^, with q < 0.05 considered significant.

To assess whether clinical and treatment heterogeneity accounted for between-subject differences in ΔPSC, we fit cluster-wise robust linear regression models with ΔPSC as the outcome and fixed effects for posterior fossa syndrome (PFS; yes/no), CSI dose (Gy; continuous), tumor location group (midline/intraventricular versus hemispheric/CPA), sex, and age at irradiation (mean-centered). For each covariate, p-values were adjusted across clusters using Benjamini–Hochberg FDR (q < 0.05).

### Signature Expression and multiregional activation-contrast score (MACS) Analyses

To bridge multivariate decoding with clinically interpretable effect metrics, we derived two within-subject scalar measures: (i) an out-of-fold classifier signature expression score and (ii) a predefined MACS in PSC units.

#### Signature expression score (SVM decision function).

In each LOSO fold, the final linear SVM (trained only on the training subjects, using the searchlight-derived mask and recursive feature elimination subset determined exclusively within the training data) was applied to the held-out subject’s TP1 and TP2 scans to obtain the SVM decision function value, also known as decision value (DV)^25^:

$$DV=f(x)=w^{\backslash \text{top}\backslash :}x+b$$

DVs were oriented so that higher values corresponded to the post-irradiation class (TP2). Within-subject change was defined as Δ *DV* = *DV*_TP2_ − *DV*_TP1_. We tested whether ΔDV differed from zero using a two-sided Wilcoxon signed-rank test and reported effect size $r=\frac{\left|z\right|}{\sqrt{N}}$. For descriptive reporting, we report the percentage of subjects with Δ *DV* > 0. As a nonparametric confirmation, we performed a within-subject label-swap (sign-flip) permutation test on mean(ΔDV) using 20,000 iterations.

#### Multiregional activation-contrast score (MACS).

After identifying the final set of clusters from the multivariate feature selection results (see above), clusters (*k*) were partitioned into two sets according to the sign of their cohort median within-subject change ΔPSC: *𝒞*_+_ (clusters with positive median ΔPSC) and *𝒞*_−_ (clusters with negative median ΔPSC). We then defined a multiregional activation-contrast score (MACS) for each timepoint (tp) as:

$$\text{MACS}(tp)=\frac{1}{K_{+}}\sum_{k\in {𝒞}_{+}}PSC_{k}(tp)-\frac{1}{K_{+}}\sum_{k\in {𝒞}_{-}}PSC_{k}(tp),$$

Where *PSC_k_* (*tp*) is the cluster-wise percent signal change at timepoint, while *K*_+_ and *K*_−_ are the numbers of clusters in the *𝒞*_+_ and *𝒞*_−_, respectively. Within-subject change was defined as Δ *MACS* = *MACS*_TP2_ *− MACS*_*TP*1_ and tested against zero using a two-sided Wilcoxon signed-rank test with effect size r and percentage positive ΔMACS.

#### Association between metrics.

We assessed concordance between ΔDV and ΔMACS using Spearman’s rank correlation.

#### Exploratory five-timepoint longitudinal analysis (MACS trajectory).

An exploratory analysis evaluated MACS trajectories across TP1–TP5 in the subset of participants with complete five-visit data (n = 8). MACS was computed at each timepoint using the same cluster definition described above. We tested the overall effect of timepoint using a linear mixed-effects model with timepoint as a categorical fixed effect and a random intercept for subject, restricted maximum likelihood (REML) approach^26^. Post hoc, we compared each later timepoint (TP2–TP5) against TP1 using paired Wilcoxon signed-rank tests and applied Benjamini–Hochberg FDR correction across these four comparisons. Because Cogmed participation was not uniform and only 2 out of 8 participants in this TP1–TP5 subset received computerized training, we did not interpret TP4 as a Cogmed intervention effect; TP3–TP5 are treated as protocol-defined follow-up visits.

## Results

**Figure 1:**
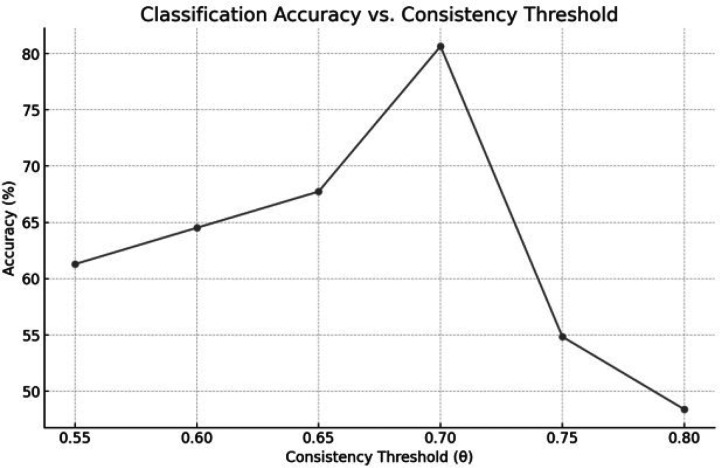
Leave-one-subject-out classification accuracy of a linear SVM separating TP1 (pre-radiotherapy) vs TP2 (early post-radiotherapy) scans using whole-brain voxelwise task-versus-rest activation t-maps from silent verb generation fMRI (n=31 paired subjects). The consistency thresholds (θ) is the minimum fraction of training subjects that must share the same sign of within-subject activation change (Δt = t_TP1 - t_TP2) for a voxel to be retained in the stage-1 sign-consistency filter (followed by searchlight ranking and SVM recursive feature elimination). Accuracy peaked at θ=0.70 (80.7%).

The LOSO cross-validation revealed a non-monotonic relation between the sign-consistency threshold (θ) and decoding accuracy. Overall cross-validated accuracy increased steadily from θ = 0.55 (61%) to θ = 0.70, where it peaked at 80.7% (Fig. 1). Beyond this optimum, performance deteriorated sharply: θ = 0.75 yielded accuracy slightly above chance (54.8%), and θ = 0.80 fell to 48.4%. Sensitivity and specificity mirrored overall accuracy at every threshold because each test fold comprised exactly one TP1 (pre-irradiation) and one TP2 (post-irradiation) scan.

**Figure 2:**
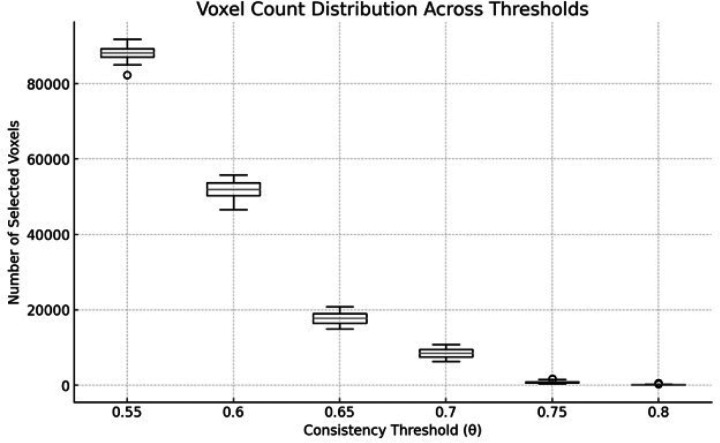
Distribution of the number of voxels entering the classifier at each sign-consistency threshold (θ) across leave-one-subject-out folds. Voxels were retained only if the within-subject activation change (Δt = t_TP1 - t_TP2) had the same sign in at least θ of the training subjects (stage 1), then reduced by searchlight ranking and SVM recursive feature elimination (stage 2). Boxplots summarize per-fold feature counts, illustrating the trade-off between enforcing cross-subject consistency and retaining sufficient features.

Concomitantly, the number of voxels retained after the two-stage feature-selection pipeline collapsed exponentially with more stringent thresholds (Fig. 2). Median feature count shrank from ~ 87 k voxels at θ = 0.55 to ~ 8 k voxels at θ = 0.70 and to < 200 voxels at θ = 0.80. The accuracy peak therefore depended on the balance between group-level consistency (high θ) and information sufficiency (adequate feature dimensionality).

Collectively, these results demonstrate that enforcing a moderate (70%) sign-consistency criterion maximizes generalizable discriminative power while retaining a neuroanatomically interpretable subset of voxels. At the optimal consistency threshold (θ = 0.70), we assessed performance with leave-one-subject-out cross-validation and a paired pre-to-post label-swap permutation test (n = 200) that preserves within-subject structure. Observed accuracy under the true labels (80.7%) was compared with the randomized-classification null mean of 50.16% (95% null confidence interval: 29.03–72.58%). The improvement was significant (one-sided permutation p = 0.0149; Cohen’s d = 2.92). Therefore, the cross-validated accuracy is significantly higher than that of randomized classification.

**Figure 3:**
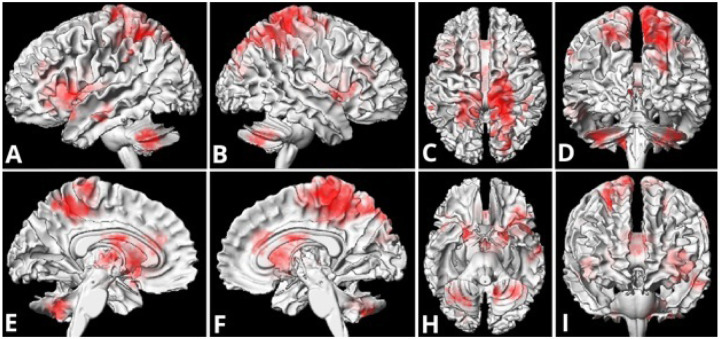
Spatial distribution of informative voxels at the optimal consistency threshold (θ = 0.70) for the consistency-aware search-light feature selection. A voxel appears overlaid in red if it was retained as a feature in at least one leave-one-subject-out fold. Panels show 3D brain surface render views (A: left lateral; B: right lateral; C: superior; D: posterior; E: left medial; F: right medial; H: inferior; I: anterior). Left lateral and anterior (A, I) show left inferior frontal gyrus (pars opercularis/triangularis; Broca’s region) and anterior insula, key for lexical retrieval and articulatory planning^29,30^. Medial views (E, F) demonstrate the supplementary motor area and anterior cingulate/medial frontal cortex supporting selection and internal speech^31,32^, with involvement of the medial precuneus. The superior and posterior views (C, D) highlight the superior parietal lobule, precuneus, paracentral and postcentral regions, which are phonological and attentional components of verb generation^33,34^. Inferior view (H) emphasizes the bilateral cerebellar Crus I/II and lobules VI–VIII, which are territories of the cerebello-frontal language loop^34^ and territories highly exposed during craniospinal and posterior-fossa irradiation.

Moreover, still at the performance-optimal threshold (θ = 0.70), the informative features formed a distributed, neuroanatomically interpretable pattern (Table 2) encompassing canonical language and control circuitry^29–33^ along with cerebellar territories^34^ commonly implicated in language–motor integration (Fig. 3).

Within-subject ΔPSC analyses revealed statistically robust parietal changes. The “right precuneus/superior parietal/paracentral/postcentral” cluster showed attenuation of task-negative response (mean ΔPSC = + 0.323, median ΔPSC = + 0.252; Wilcoxon signed-rank p = 0.00239, q = 0.00835, r = 0.55). The “left supramarginal/inferior parietal” cluster showed increased activation (mean ΔPSC = + 0.338, median ΔPSC = + 0.211; p = 0.000650, q = 0.00455, r = 0.61). Other clusters (including bilateral cerebellum; left inferior frontal gyrus, insula, and basal ganglia; anterior cingulate; and left middle temporal gyrus) showed negative mean ΔPSC but did not survive FDR correction (lowest q = 0.0509 for the left cerebellar cluster). In sensitivity analyses adjusting for covariates, ΔPSC was not significantly associated with PFS history, CSI dose, tumor location group, sex, or age at irradiation, either in uncorrected tests (all p values > 0.05) or after false discovery rate (FDR) correction (all q values > 0.05).

In the cerebral cortex, decreased activation was observed in the left inferior frontal gyrus (pars opercularis/triangularis) and the anterior insula, regions implicated in lexical retrieval and articulatory planning. Medial frontal contributions included supplementary motor area and anterior cingulate cortex, consistent with internal speech generation, response selection, and performance monitoring. Parietal engagement comprised superior parietal lobule and precuneus, with extension into paracentral/postcentral cortex, aligning with phonological working memory and attentional components of verb generation.

Subcortically, there was robust bilateral involvement of cerebellar Crus I/II and lobules VI–VIII, which are territories of the cerebro-cerebellar language loop that are highly exposed during craniospinal and posterior fossa irradiation^35^.

Taken together, these spatial findings support the interpretation that post-irradiation reductions in task-related activation occur within a cerebello-frontal–temporal network subserving language production and cognitive control, and that a moderate sign-consistency criterion preferentially captures these treatment-sensitive regions.

### Signature expression score (SVM decision function).

Out-of-fold SVM DV, oriented so that higher values corresponded to TP2 (post-irradiation) class, showed a consistent within-subject shift from TP1 to TP2. Across 31 subjects, Δ *DV = DV*_TP2_ *− DV*_*TP*1_ was positive in 25/31 (80.6%). Median ΔDV = 0.846 (IQR 1.598) and mean ΔDV = 0.953. The paired shift was significant by Wilcoxon signed-rank (p = 0.000650; r = 0.612) and remained significant in a within-subject label-swap (sign-flip) permutation test on mean(ΔDV) (two-sided p = 0.0003; 20,000 iterations).

### Multiregional activation-contrast score (MACS).

The PSC-unit MACS, defined as the mean PSC across clusters with positive cohort median Δ *PSC*(*𝒞*_+_) minus the mean PSC across clusters with negative cohort median Δ *PSC*(*𝒞*_−_), increased from TP1 to TP2 in 30/31 (96.8%) subjects. In this cohort, *𝒞*_+_ comprised clusters on parietal regions, while *𝒞*_−_comprised clusters on cerebellar, frontal, insula, and cingulate regions (see Table 2 for reference values). Within-subject change Δ *MACS = MACS*_*TP*2_ *− MACS*_TP1_ had median 0.364 (IQR 0.358) and mean 0.453, being significant by Wilcoxon signed-rank (p = 2.0×10^−^^6^; r = 0.859).

### Association between metrics.

Δ *MACS*correlated with Δ *DV* (Spearman ρ = 0.514, p = 0.0031), indicating that the clinically interpretable activation-contrast captured a substantial portion of the multivariate signature expression.

### Exploratory five-timepoint MACS trajectory.

**Figure 4:**
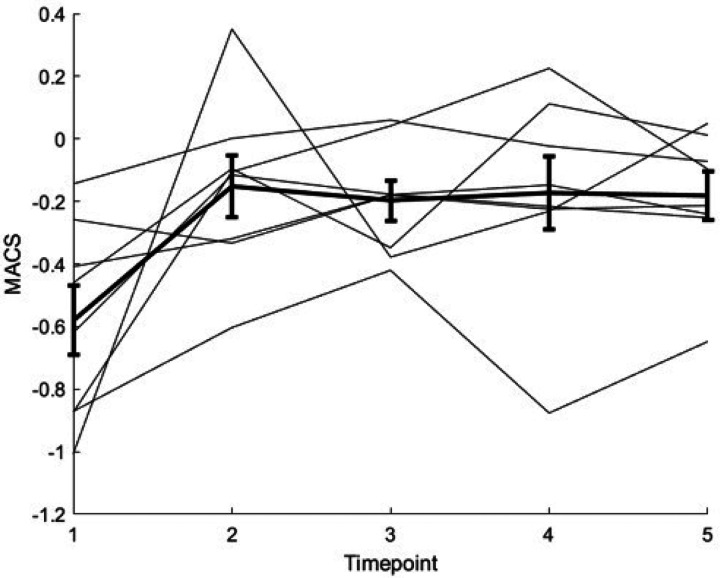
Exploratory Multiregional Activation-Contrast Score (MACS) trajectory across TP1–TP5 (n=8). Thin lines show individual participants; thick line shows mean ± SE. Timepoints are protocol-defined: TP1 pre-irradiation, TP2 post-irradiation/pre-chemotherapy, TP3 post-chemotherapy, TP4 protocol follow-up visit scheduled after the Cogmed trial period (Cogmed participation were restricted to 2 out of these 8 participants), and TP5 6-month follow-up after TP4. MACS (in percentage signal change units) increased from TP1 to TP2 and remained elevated through TP5, consistent with a durable multiregional activation shift beyond the immediate post-irradiation window.

In the subset of participants with complete five-visit data (n = 8), MACS showed a significant overall timepoint effect (linear mixed-effects model with timepoint as categorical fixed effect and subject random intercept: p = 0.001366). Relative to TP1 (pre-irradiation), MACS was higher at TP2 (post-irradiation/pre-chemotherapy; median ΔMACS = 0.316; p = 0.015625; 7/8 positive), TP3 (post-chemotherapy; median ΔMACS = 0.336; p = 0.007812; 8/8 positive), TP4 (protocol follow-up visit scheduled after the Cogmed trial period; median ΔMACS = 0.332; p = 0.015625; 7/8 positive), and TP5 (6-month follow-up after TP4; median ΔMACS = 0.295; p = 0.007812; 8/8 positive). Because only 2 out of 8 participants received Cogmed training, TP4 should not be interpreted as a post-Cogmed effect. All four comparisons remained significant after FDR correction (q = 0.015625). The trajectory showed an early upward shift from TP1 to TP2 that persisted through TP5 (Fig. 4).

## Discussion

Our findings corroborate the established concern that pediatric radiotherapy can lead to alterations in functional brain activation, potentially leading to cognitive deficits.^36^

The machine-learning pipeline identified a distributed signature spanning cerebellar, frontal, medial, and parietal regions. When within-subject change in these clusters was quantified using ΔPSC (TP2 – TP1), the most statistically robust effects were parietal: attenuation of pre-treatment task-negative response in the right precuneus and superior parietal cluster and increased activation in the left supramarginal and inferior parietal cluster (Wilcoxon signed-rank q < 0.01; 71–81% of participants showing increases). In contrast, mean decreases were observed in cerebellar and left fronto-temporal, insular, cingulate clusters; however, these showed substantial individual variability and did not survive FDR correction. Importantly, lack of FDR significance for individual clusters does not imply irrelevance for decoding: multivariate classifiers can leverage weak and/or heterogeneous regional effects when they combine with other features and covariance structure. Accordingly, we interpret these cerebello–fronto–insular and cingulate regions as components of a distributed multivariate signature rather than as stand-alone univariate effects.

Reductions in task-evoked BOLD response in classical cortical language regions, particularly the inferior frontal gyrus and the left middle temporal gyrus, may reflect less efficient recruitment of controlled lexical–semantic retrieval and selection processes during covert verb generation.^29,30^ Given potential post-radiotherapy alterations in neurovascular coupling, these changes may also partly reflect hemodynamic responsivity rather than purely neuronal effects. Moreover, these findings indicate that treatment-related changes in task-evoked activation during silent verb generation are not confined to a single perisylvian “language center,” but instead involve a distributed network spanning frontal, temporal, parietal, and cerebellar territories commonly implicated in lexical retrieval, articulatory planning, phonological buffering, and attentional control.^29^ Because these regional decreases showed substantial inter-individual variability and did not survive FDR correction, they are best interpreted as components of the multivariate signature rather than as stand-alone univariate effects.

At the performance-optimal sign-consistency threshold (θ = 0.70), features with the greatest discriminative value localized to left inferior frontal gyrus (pars opercularis and triangularis) and anterior insula; medial-frontal control regions including supplementary motor area and anterior cingulate; dorsal parietal and precuneus regions with extension into paracentral/postcentral cortex; and bilateral cerebellar Crus I/II and lobules VI–VIII.

This anatomy is compatible with the cerebellar-cerebral language loop and suggests that radiation exposure, particularly to posterior fossa structures and their thalamo-cortical projections, may perturb an extended production and control circuit rather than a narrow cortical module. Notably, the patterned (but individually variable) decrease in the cerebellar response during the silent verb generation task for patients after radiotherapy aligns with growing evidence that cerebellar Crus I/II and lobules VI–VIII contribute to higher cognitive operations, including language production, articulation coordination, and executive sequecing.^37–39^

The increased engagement in the left supramarginal gyrus and inferior parietal lobule likely reflects a greater reliance on dorsal-stream phonological mechanisms to support covert production demands when core production and control regions (left inferior frontal gyrus, insula, anterior cingulate cortex, cerebellum) show reduced task-evoked responses. The left supramarginal gyrus and inferior parietal lobule are classically implicated in the phonological store^40^ and sublexical grapheme-phoneme coding^41^, with projections via superior longitudinal fasciculus^42^ and arcuate fasciculus^43^ to premotor cortex and inferior frontal gyrus, supporting articulatory rehearsal during covert speech. Because this effect persists when pooling auditory and visual cueing and primary sensory cortices are not consistent contributors, it is less likely to be sensory-driven; instead, it appears modality-independent, indexing increased phonological working-memory load. Although overt task performance was not directly measured during scanning, this pattern is compatible with compensatory recruitment of parietal phonological buffering systems. Clinically, this motivates targeting phonological rehearsal strategies and working-memory scaffolds to harness supramarginal gyrus and inferior parietal lobule recruitment, consistent with prior trials of cognitive working-memory training (e.g., Cogmed) in pediatric brain tumor survivors^44^.

The shift from task-negative at pre-irradiation toward a near-zero response post-irradiation in the right hemisphere indicates attenuation of task-negative activity in a large cluster overlapping medial parietal (precuneus) and dorsal parietal/somatomotor territories. This pattern is consistent with weaker suppression of default-mode processes at medial parietal regions (precuneus) during task engagement, which has been linked to reduced processing efficiency and greater intrusion of internally directed cognition in demanding tasks.^40,45^ In addition, reduced precuneus deactivation under demand has been interpreted as reflecting increased monitoring and control demands in some contexts^46^, although in clinical cohorts it is frequently discussed in terms of inefficient task engagement or reduced default-mode suppression. At the same time, because the cluster extends into superior parietal, paracentral, and postcentral cortices, the net ΔPSC may also reflect greater reliance on attention and sensorimotor/phonological rehearsal mechanisms (e.g., covert articulatory or somatosensory imagery) to stabilize retrieval when frontal–cerebellar components are weakened.^47^ Notably, these ΔPSC shifts were not significantly explained by PFS history, CSI dose, tumor location group, sex, or age at irradiation in covariate sensitivity analyses, although larger cohorts will be required to test moderation effects with adequate power.

Beyond classification accuracy, the out-of-fold signature expression decision values provide a patient-level measure of the distributed multivariate effect, showing a consistent TP1 to TP2 shift in most participants. Complementarily, the PSC-unit MACS (mean of the parietal clusters minus the mean of the remaining clusters) showed a highly consistent increase, supporting a robust rebalancing toward parietal recruitment during silent verb generation after radiotherapy. The correlation between these metrics suggests that a simple contrast in PSC units captures meaningful variance in the multivariate signature while remaining clinically interpretable.

TP2 scans showed reduced task-evoked activation in the supragenual anterior cingulate cortex, a medial frontal region implicated in performance monitoring and selection during covert speech.^48,49^ However, a cross-sectional sample of task fMRI alone cannot determine whether this observed change reflects direct tissue injury, vascular effects, or compensatory reorganization. A potential concern in early post-RT fMRI is that observed differences could reflect transient physiological or behavioral effects rather than durable reorganization. The exploratory TP1–TP5 analysis helps address this: in the complete five-visit subset (n = 8), MACS increased immediately after irradiation (TP2, prior to chemotherapy) and remained elevated at later protocol-defined visits through TP5, including post-chemotherapy and 6-month follow-up (omnibus LME p = 0.0014; all TP2–TP5 vs TP1 q = 0.0156). Although later phases include additional exposure (chemotherapy), the persistence of the activation-contrast supports the interpretation that the early post-irradiation shift is not purely transient and may reflect a stable change in the balance between parietal engagement and cerebello–fronto–insular and cingulate components of the signature.

Regarding the practical implications, the out-of-fold SVM DV (signature expression) and the PSC-unit MACS provide complementary, continuous biomarkers that can be tracked longitudinally in individual patients. In this cohort, both measures showed a significant within-subject post-irradiation shift (ΔDV > 0 in 25/31; Wilcoxon signed-rank p = 0.000650, r = 0.612; sign-flip permutation p = 0.0003; ΔMACS > 0 in 30/31; Wilcoxon signed-rank p = 2.0×10^−^^6^, r = 0.859), supporting an interpretable basis for imaging-based surveillance: longitudinal deviations of these scalar metrics could flag atypical functional trajectories or accumulating treatment burden before overt behavioral decline.

However, clinical scalability will depend on streamlining acquisition (brief, motion-robust paradigms feasible across ages), automating preprocessing, and demonstrating cross-scanner reproducibility and incremental value beyond standard surveillance MRI; therefore, near-term use is most realistic within prospective trials or targeted high-risk subgroups. Finally, the anatomical pattern points to plausible targets for early intervention (e.g., language therapy emphasizing lexical retrieval and phonological rehearsal; supports for verbal attention and working memory; school accommodations that offload working-memory demands) and, cautiously, motivates future investigation of neuromodulatory strategies^50,51^ aimed at frontal–cerebellar circuitry.

Methodologically, the non-monotonic accuracy profile across thresholds clarifies an important trade-off in feature selection for pediatric longitudinal fMRI: as θ increases, voxels with inconsistent group-level signs are excluded, improving noise robustness; yet overly stringent thresholds collapse feature dimensionality and remove informative but moderately variable regions. The peak at θ = 0.70 likely reflects a sweet spot balancing group-level reliability with sufficient coverage to capture treatment-sensitive signal across cerebello-frontal pathways. LOSO cross-validation with balanced classes further explains the lockstep behavior of sensitivity and specificity and supports generalizability across individuals. To guard against overfitting, we also compared the LOSO performance to a randomized baseline using a paired TP1–to–TP2 label-swap permutation test (n = 200) that preserves within-subject structure, confirming that the observed 80.7% accuracy was significantly above chance (null mean 50.16%, 95% CI [29.03%, 72.58%], one-sided p = 0.0149; Cohen’s d = 2.92).

The silent verb generation task proved effective for identifying functional changes that might not be evident using standard clinical assessments alone. Given that pediatric patients often compensate for deficits early in the recovery trajectory, the observed fMRI changes are a signal for clinicians to anticipate later impairments.^6,52^ Early interventions that directly target the implicated mechanisms, such as language therapy emphasizing lexical retrieval and phonological rehearsal, structured working-memory training, and, in emergent readers, early structured reading intervention (e.g., phonological awareness and phonics-based instruction), could be explored in a trial setting to preserve or restore function.^53^

Moreover, individualized radiation planning may leverage advanced techniques (e.g., intensity-modulated radiation therapy) to reduce dosage to vulnerable structures.^54^ Preserving cerebellar integrity and frontal-temporal pathways could mitigate neurocognitive decline.^55^ This rationale underscores an urgent need for integrative protocols combining imaging-based risk stratification with biologically informed therapeutic strategies.

Regarding limitations that warrant consideration, the typical sample sizes in pediatric oncology fMRI studies reduce power for detailed region-by-region analysis. Additionally, the heterogeneous clinical courses (e.g., chemotherapy regimens, dose distributions, and time since treatment) may introduce variability that our design cannot fully address. Motion and arousal, despite standard controls, remain potential confounds in child cohorts. Although the classifier was tuned and evaluated with LOSO cross-validation, an external test set and prospective acquisition would strengthen claims of generalizability. Finally, while task fMRI identifies treatment-sensitive regions, it does not by itself specify whether reduced activation reflects neuronal inefficiency, compensatory reorganization, or vascular changes. In addition, although signature expression scores are computed out-of-fold, the spatial signature and MACS definition were derived in this cohort; replication in independent samples and association with prospective neurocognitive outcomes are needed. Finally, because only 2 of the 8 participants in the TP1–TP5 subset received the Cogmed intervention, we cannot evaluate cognitive-training effects based on this TP1–TP5 subset.

This approach could be refined by integrating additional features, such as diffusion tensor imaging (DTI) metrics, resting-state connectivity, or volumetric measures, to create a more comprehensive biomarker panel.^21,56–59^ Such biomarkers, once validated, could inform early interventions (e.g., cognitive rehabilitation, pharmacological approaches) to ameliorate or prevent significant functional changes.^6,60^ The same multimodal biomarker framework could also provide mechanistic readouts and sensitive endpoints for evaluating neuroprotective agents, such as memantine, by quantifying whether treatment preserves task-evoked network engagement and white-matter integrity over time.

Future work should integrate diffusion metrics, resting-state connectivity, and volumetry with task activation to sharpen mechanistic interpretation and improve predictive accuracy. Dose-mapping against discriminative features could clarify dose–response relationships along cerebello-thalamo-cortical tracts. Longitudinal trajectories spanning acute, subacute, and late effects, paired with language and cognitive assessments will be essential for linking imaging biomarkers to functional outcomes and for personalizing supportive care. Importantly, this framework could also serve as an objective endpoint for intervention trials by testing whether cognitive training and rehabilitation are accompanied by pre- to post-intervention changes (normalization or strengthening) in functional connectivity and task-evoked engagement within the identified circuit, and whether those neural shifts track behavioral gains.

## Conclusion

This longitudinal analysis shows that a consistency-aware machine-learning approach can distinguish between pre- and post-radiotherapy silent verb generation fMRI with high accuracy while retaining an interpretable multiregional signature. Patient-level bridge metrics captured the distributed effect: out-of-fold SVM decision function values showed a systematic within-subject shift (median ΔDV = 0.846; p = 0.000650; sign-flip permutation p = 0.0003), and the PSC-unit multiregional activation-contrast score increased in 30/31 subjects (median ΔMACS = 0.364; p = 2.0×10^−^^6^). In an exploratory TP1–TP5 subset (n = 8), MACS increased after irradiation (TP2, prior to chemotherapy) and remained elevated post-chemotherapy and through 6-month follow-up (LME p = 0.0014; TP2–TP5 vs TP1 q = 0.0156), supporting durability beyond the immediate post-irradiation window. Together, these findings provide quantitative, clinically interpretable biomarkers that motivate longitudinal imaging-based surveillance and targeted interventions to preserve language and cognitive function.

## Figures and Tables

**Table 1:** Demographic and clinical characteristics of the medulloblastoma cohort (n = 31). CSI = craniospinal irradiation; PFS = posterior fossa syndrome. Postoperative neurologic categories are minimal symptoms, PFS1 complete mutism, PFS2 reduced speech, and severe ataxia. Cerebellopontine angle (CPA) tumors were grouped with hemispheric tumors.

Participant demographic and clinical characteristics	Overall (n = 31)
Age at irradiation, years	14.1 ± 4.7
Sex, n (%)	
Male	18 (58.1)
Female	13 (41.9)
Posterior fossa syndrome history, n (%)	
Yes	8 (25.8)
No	23 (74.2)
Postoperative neurologic categories, n (%)	
Minimal neurological symptoms after surgery	22 (71.0)
PFS1 (complete mutism)	3 (9.7)
PFS2 (reduced speech)	5 (16.1)
Severe ataxia	1 (3.2)
Tumor location, n (%)	
Midline/intraventricular	21 (67.7)
Hemispheric/CPA	10 (32.3)
Craniospinal irradiation dose, n (%)	
15 Gy	5 (16.1)
23.4 Gy	10 (32.3)
33 Gy	1 (3.2)
≥36 Gy	15 (48.4)
Primary tumor bed boost dose, n (%)	
51.0 Gy	6 (19.4)
54.0 Gy	24 (77.4)
59.4 Gy	1 (3.2)
Radiation modality, n (%)	
Photon	14 (45.2)
Proton	9 (29.0)
Mixed	8 (25.8)

**Table 2:** Cluster-wise changes in the silent verb-generation task–evoked blood-oxygen-level-dependent (BOLD) signal. Clusters were defined as the union of voxels selected by a sign-consistency–based machine-learning model across leave-one-subject-out (LOSO) folds at threshold θ = 0.70; only components with ≥ 100 voxels are reported. For each cluster, the center of gravity is given in MNI coordinates, and anatomical descriptors are provided using the full region names from the Automated Anatomical Labeling^27^ atlas (AAL). Group-mean BOLD percent signal change^28^ (PSC) is reported for TP1 (pre-radiotherapy) and TP2 (post-radiotherapy) visits. ΔPSC denotes ‘TP2 – TP1’; arrows indicate the direction of change (↑ increase, ↓ decrease). Cluster size is the number of voxels in the connected component.

Cluster labels	Hemisphere	Gravity Center	Cluster Size	Mean	PSC	ΔPSC	Direction
(ALL)		(MNI)	(Voxels)	(TP1)	(TP2)	(TP2 - TP1)	
Cerebellum (Crus I, Lobule VIII, Lobule VI, Crus II)	R	30, −63, −39	542	0.371	0.293	−0.078	↓
Cerebellum (Crus I, Lobule VIII, Lobule VI, Crus II)	L	−26, −58, −39	940	0.294	0.115	−0.179	↓
Middle Temporal Gyrus	L	−55, −15, −14	280	0.179	0.133	−0.046	↓
Insular Cortex, Putamen, Inferior Frontal Gyrus (triangular part)	L	−12, 8, 5	5134	0.515	0.381	−0.134	↓
Anterior Cingulate Cortex (Supragenual)	Midline	1, 32, 22	1076	0.345	0.205	−0.140	↓
Precuneus, Superior Parietal Lobule, Paracentral Lobule, Postcentral Gyrus	R	9, −44, 56	7633	−0.353	−0.030	0.323	↑
Supramarginal Gyrus, Inferior Parietal Lobule	L	−62, −43, 37	181	−0.001	0.337	0.338	↑

## Data Availability

Data supporting the findings of this study can be made available by the corresponding author upon reasonable request and with appropriate institutional data use agreements.

## Key Points

1. Silent verb-generation fMRI shows early post-RT cerebellar-cerebral changes.
2. Linear SVM separated pre vs post RT scans (LOSO 80.7% accuracy; p=0.015).
3. Key regions: cerebellar Crus I/II, IFG/insula, cingulate, temporal, parietal.

## Importance of the Study

This study identifies critical changes in language-related brain activation in pediatric medulloblastoma patients who underwent radiotherapy. Using a silent verb generation fMRI paradigm, we identify a multivariate, anatomically interpretable post-RT signature that includes cerebellar, frontal, insular, and cingulate regions, as well as temporal and parietal language control regions, with robust parietal PSC shifts and trend-level decreases across other signature components. These findings underscore the cerebellum’s largely underappreciated influence on language networks and extend our understanding of how radiotherapy disrupts neural circuits subserving higher-order cognitive processes. Demonstrating the classification of imaging data using machine learning methods presents a promising avenue for early detection of treatment-induced neurocognitive risks and can guide targeted cognitive rehabilitation strategies. Our results support longitudinal functional monitoring, where feasible, to detect and help manage radiation-related impairments and thereby to improve quality of life in patients with medulloblastoma and survivors.
